# Merkel cell carcinoma in the context of follicular lymphoma treated with radiation and avelumab

**DOI:** 10.1016/j.jdcr.2024.03.027

**Published:** 2024-04-27

**Authors:** Aya Nishizawa, Misaki Kawakami, Moe Makiguchi, Akihito Ookunitani, Keisuke Goto

**Affiliations:** a Department of Dermatology, Tokyo Metropolitan Cancer and Infectious Disease Center, Komagome Hospital, Tokyo, Japan; b Department of Pathology, Tokyo Metropolitan Cancer and Infectious Disease Center, Komagome Hospital, Tokyo, Japan

**Keywords:** avelumab, follicular lymphoma, Merkel cell carcinoma, radiation

## Introduction

Merkel cell carcinoma (MCC) is a rare and aggressive neuroendocrine cancer of the skin with a high recurrence rate and a tendency to spread rapidly to lymph nodes and distant organs. Risk factors include long-term ultraviolet exposure, Merkel cell polyomavirus infection, immunosuppression, and hematologic malignancies such as chronic lymphocytic leukemia.^1^, ^2^, ^3^ Follicular lymphoma (FL) is generally a low-grade lymphoma. The occurrence of relapse characterizes the natural history of FL and histologic transformation into an aggressive lymphoma, which is expected to occur at a rate of 2% to 3% each year.^4^ FL is characterized by diffuse lymphadenopathy, bone marrow involvement, and splenomegaly; cutaneous lesions may also be present. Herein we report a case of cooccurring FL and MCC with skin, lymph node, and bone marrow involvement presenting challenges to accurate diagnosis and treatment.

## Case report

**Fig 1:**
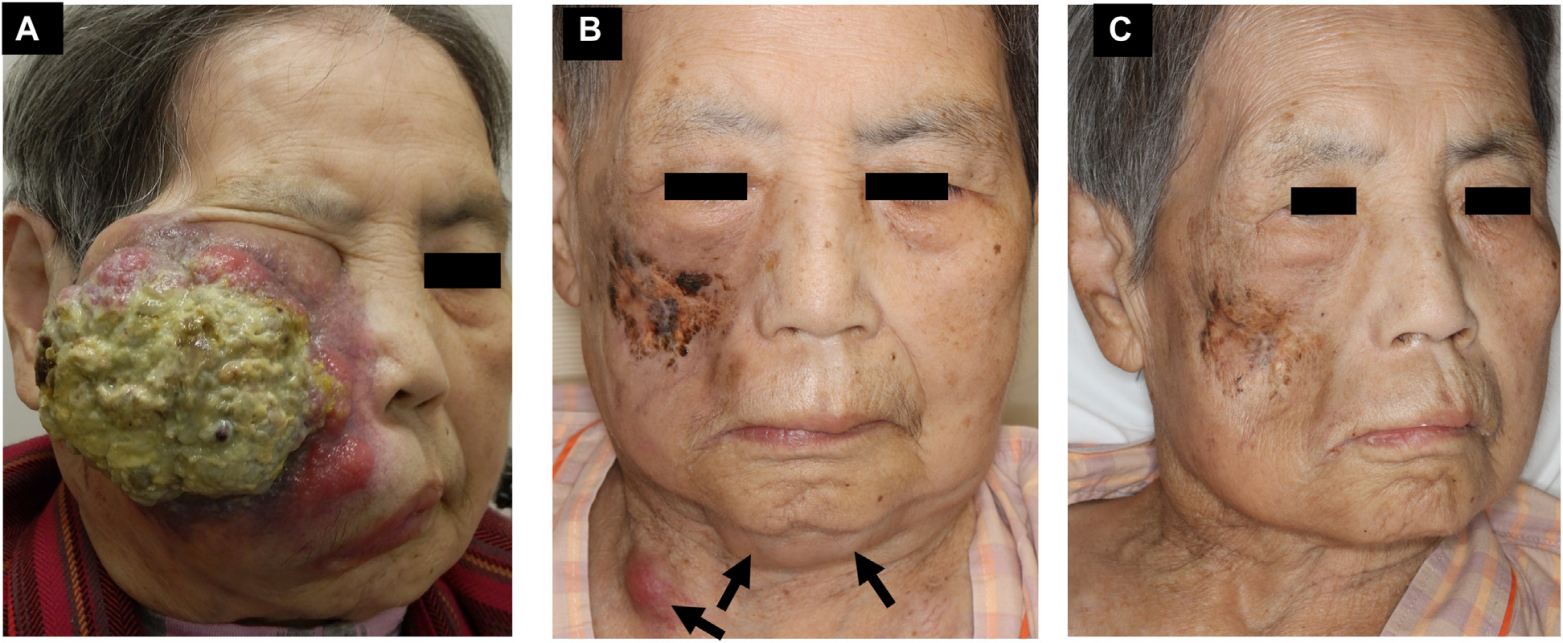
Clinical appearance. **A,** Clinical appearance at the initial examination. **B,** Clinical appearance of the Merkel cell carcinoma recurrence. A 6 cm, subcutaneous mass can be seen on the mandible, and a *red* nodule can be seen on the neck. **C,** Clinical appearance after 4 courses of avelumab. The Merkel cell carcinoma skin metastases have disappeared. *Arrow*: Merkel cell carcinoma skin metastasis to the neck and the mandible.

An 87-year-old female patient received the diagnosis of early stage (stage 2) grade 1 FL based on a left palatal mass and sublingual biopsy 4 years ago but was placed on observation due to her advanced age and lack of desire for treatment. She presented to the dermatology department with a 5-month history of a rapidly growing, painful nodule on the right cheek. On examination, she had an 11 cm erythematous mass that obstructed the right eye and mouth from fully opening (Fig 1, *A*).

**Fig 2:**
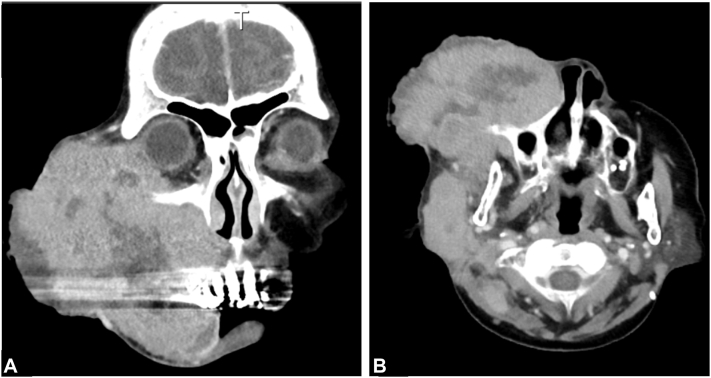
Computed tomography of the primary Merkel cell carcinoma lesion (right cheek) at the initial examination. **A,** Coronal view. **B,** Axial view.

**Fig 3:**
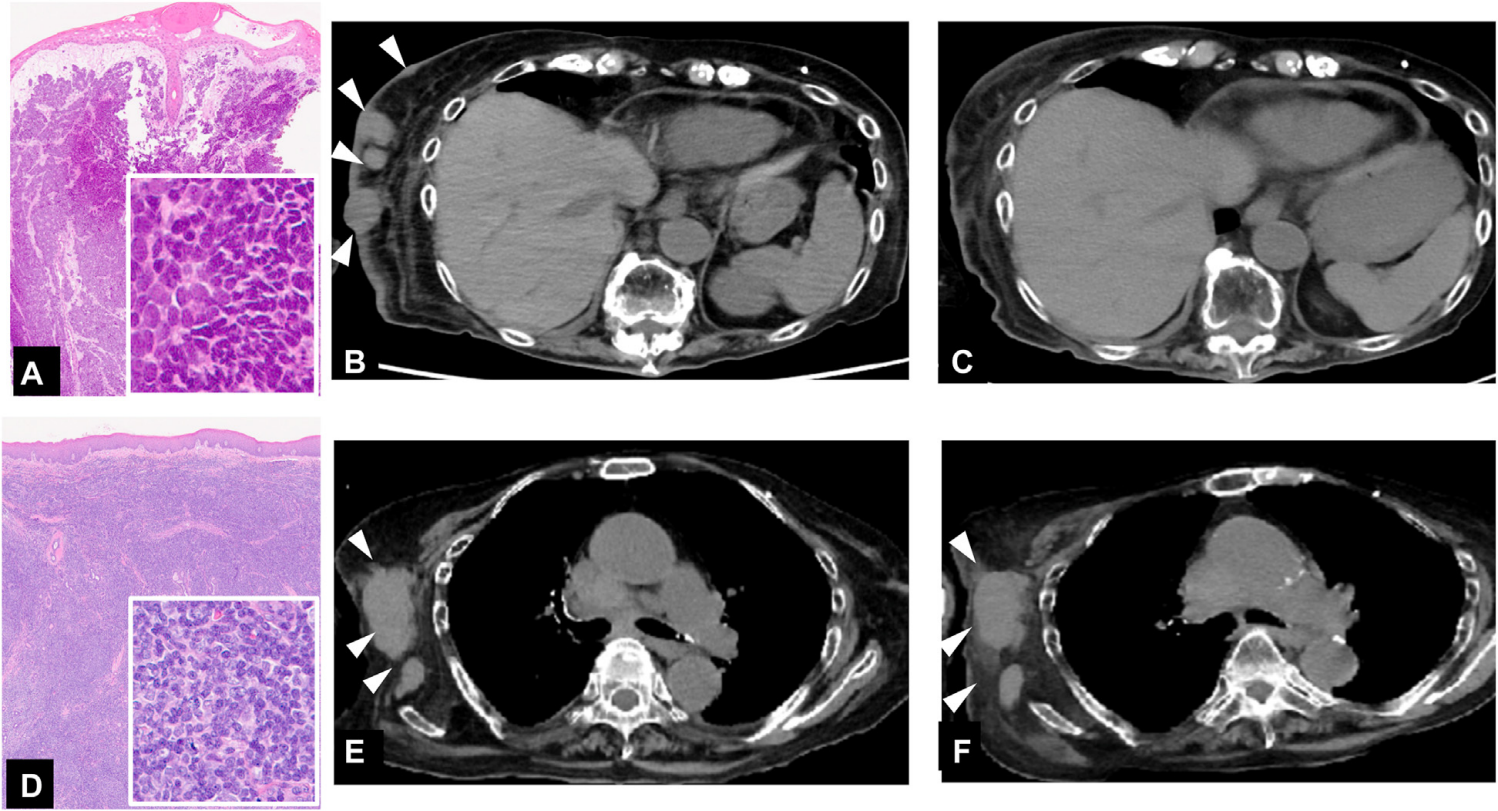
Histopathology of the biopsy specimens and treatment course of metastatic Merkel cell carcinoma and follicular lymphoma. **A,** Biopsy specimen from the right cheek mass demonstrated a sheet-like growth of small tumor cells with finely granular round nuclei and numerous, mitotic elements within the stroma. **B,** Computed tomography of the Merkel cell carcinoma metastases to the skin on the trunk before treatment with avelumab. **C,** Computed tomography of the Merkel cell carcinoma metastases to the skin of the trunk after treatment with avelumab. **D,** Biopsy specimens of the right axillary lymph node. H&E-stained sections of the bilateral cervical lymph nodes demonstrated a proliferation of medium to large, atypical lymphocytes forming follicular nodular structures. **E,** Computed tomography of a right axillary lymph node with a follicular lymphoma lesion before treatment with avelumab. **F,** Computed tomography of the right axillary lymph node with a follicular lymphoma lesion after treatment with avelumab. *Arrowheads* of (**B)** Merkel cell carcinoma metastases to the skin. *Arrowheads* of (**E,F)** lymph node with a follicular lymphoma lesion.

A blood test revealed decreased Hb at 11.1 g/dl and elevated serum lactate dehydrogenase at 1170 U/L (154-474). Serum calcium was normal at 8.8 mg/dl (8.8-10.1). Computed tomography demonstrated a 75 × 70 × 116-mm mass on the right face with deep orbital invasion (Fig 2, *A* and *B*). Lymphadenopathy was observed in multiple lymph nodes above the diaphragm (right submandibular region, bilateral neck, bilateral supraclavicular fossa, mediastinum, and right axilla). Biopsies were performed in several locations to assess for FL recurrences or metastases. Histologically, a biopsy specimen from the right cheek mass revealed a sheet-like growth of small tumor cells with finely granular, round nuclei and numerous mitotic elements within the stroma (Fig 3, *A*), which were positive for cytokeratin 20, synaptophysin, and chromogranin A. The findings indicated MCC. In contrast, the biopsy specimens of the right axillary lymph node and bilateral cervical lymph nodes revealed a proliferation of medium to large, atypical lymphocytes forming follicular, nodular structures (Fig 3, *D*). These lymphocytes were positive for cluster of differentiation 20 (CD20), cluster of differentiation 10 (CD10), B-cell/CLL lymphoma 2 (Bcl-2), Kiel 67 (Ki-67), B-cell lymphoma 6 (Bcl-6) proliferation index of 30%. These findings indicated grade IIIA FL. Both malignancies were progressive, but the MCC in particular was progressing rapidly and reducing the patient's quality of life. Moreover, because systemic treatment for the FL would lead to immunosuppression with the potential to promote MCC progression, local treatment was first administered for the MCC. After palliative irradiation 30 Gy/10 fr was administered to the mass in the right cheek, the volume of the mass significantly decreased. By the completion of the radiotherapy, the mass had flattened, and the patient was able to open her eye and mouth without difficulty.

**Fig 4:**
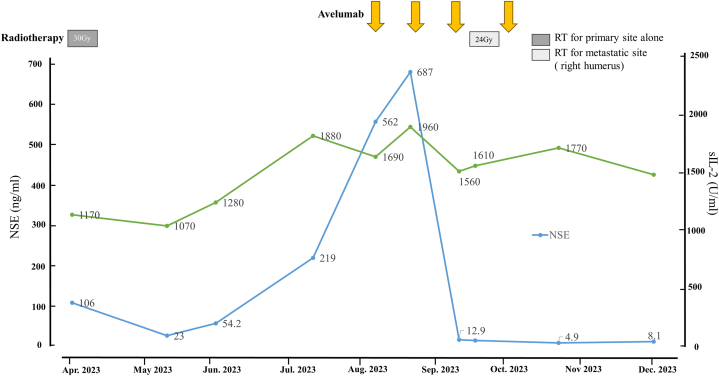
Clinical course. Treatment course and changes in serum nerve-specific enolase, a tumor marker for Merkel cell carcinoma, and serum lactate dehydrogenase, a tumor marker for follicular lymphoma. Nerve-specific enolase was elevated at the initial presentation but normalized after radiotherapy. It increased again at the time of the MCC recurrence but returned to the normal range after 2 courses of avelumab therapy. Lactate dehydrogenasedecreased slightly with avelumab treatment but remained elevated. *Arrow*, Avelumab; *NSE*, nerve-specific enolase; *RT*, radiation therapy.

No recurrence was observed on the right cheek 3 months after irradiation, but a 6 cm, subcutaneous mass appeared on the mandible (Fig 1, *B*) while multiple red nodules and subcutaneous masses appeared on the trunk. Computed tomography demonstrated further enlargement of multiple lymph nodes and new subcutaneous metastases in the trunk and neck. A zone of enhancement with evidence of bone marrow carcinomatosis was also found within the right humeral marrow. Lactate dehydrogenase had increased to 1960 U/L, and serum nerve-specific enolase had increased to 687 ng/mL from the value of 106  ng/mL obtained at the first visit (0-16.3) (Fig 4).

Biopsies of the skin nodules and the right humerus confirmed the diagnosis of MCC metastasis. The patient was restaged from II to IV and received avelumab 10 mg/kg intravenously every 2 weeks and radiation 24 Gy/6 fr to the right humerus. The MCC lesions responded well to avelumab; after 2 courses of the drug, the subcutaneous metastases showed marked reduction (Fig 1, *C*), and the nerve-specific enolase normalized (Fig 4). After 4 courses, the treatment was discontinued due to the development of pyogenic shoulder arthritis. Three months after discontinuation, computed tomography found a full resolution of the MCC and the absence of any new lesions, indicating a complete response (Fig 3, *B* and *C*). However, the FL lesions in the right axilla, neck, and mediastinal lymph nodes had decreased only slightly, indicating stable disease. The serum lactate dehydrogenase remained elevated at 1530 U/L (Figs 3, *D* and *E*, and 4).

## Discussion

In the present case, the MCC developed simultaneously with the recurrence and progression of the FL. Furthermore, when the MCC recurred and metastasized, the lymph nodes with the FL metastases also enlarged, suggesting that these 2 malignancies may have had a mutually immunosuppressive effect which contributed to their progression.

Cases of FL complicated by MCC are rare, with only 3 cases having been reported to date,^5^, ^6^, ^7^ 2 of which involved the development and rapid progression of MCC during rituximab treatment for FL.^6^^,^^7^ These reports suggested that MCC may progress due to the immunosuppressive effects on FL of chemotherapy and rituximab, which should therefore be avoided. Our patient had multiple lymphadenopathy and skin and bone marrow metastases, making it difficult to determine whether the metastases were of the FL or MCC. Thus, multiple biopsies were performed in the present case to identify the origin of the metastases and determine the best treatment strategy. Biopsies of various sites demonstrated that the lymph nodes with the FL metastases histologically had not transformed into diffuse large B-cell lymphoma, an aggressive lymphoma, and biopsies of the skin and bone marrow metastases that appeared at the time of recurrence all demonstrated MCC. Therefore, the decision was made not to treat the lymphoma but to prioritize the treatment of the MCC with local palliative irradiation, and avelumab was administered in the event of a metastatic recurrence.

Despite the high malignancy potential of MCC, spontaneous regression occurs with some frequency,^8^ and there are scattered reports of regression after biopsy.^9^ The usual curative radiation dose for primary unresectable MCC is 60 to 66 Gy.^10^ However, in the present case, the lesion almost completely resolved after 30 Gy of palliative irradiation.

The cooccurrence of MCC and hematological malignancies poses a therapeutic challenge. Both FL and MCC can involve the skin, lymph nodes and bone marrow, making diagnosis difficult on the clinical findings alone. MCC can progress rapidly with lymphoma treatments such as rituximab, so biopsies should be performed aggressively to determine the diagnosis and treatment strategy.

## Conflicts of interest

None disclosed.
